# An Integrated Statistical and Machine Learning Approach for Breast Cancer Classification Using Tumor Morphological Features

**DOI:** 10.1155/bmri/6986321

**Published:** 2026-07-10

**Authors:** Awoke Fetahi Woudneh, Nigatu Tiruneh Shiferaw, Yenesew Fentahun Gebrie, Misganaw Mekonnen Nigussie, Kebadu Tadesse Cherie, Fetene Getnet Gebeyehu

**Affiliations:** ^1^ Department of Statistics, Debre Markos University, Debre Markos, Ethiopia, dmu.edu.et

**Keywords:** AUC, breast cancer, classification, logistic regression, machine learning, random forest, support vector machine, tumor morphology

## Abstract

**Introduction:**

Breast cancer is one of the most common cancers and a leading cause of death among women worldwide. Early and accurate classification of tumors as benign or malignant is essential for improving patient outcomes. In recent years, statistical and machine learning methods have been widely used to improve diagnostic accuracy; however, combining these approaches in a single framework remains limited.

**Methods:**

A retrospective analysis was conducted using the Breast Cancer Wisconsin Diagnostic Dataset (569 tumor samples). Eight morphological features were analyzed. The dataset was split into training (70%) and testing (30%) sets using stratified random sampling. Logistic regression, random forest, and support vector machine (SVM) models were developed. Hyperparameter tuning was performed using five‐fold cross‐validation. Model performance was evaluated using accuracy, sensitivity, specificity, precision, F1‐score, Matthews correlation coefficient (MCC), and area under the curve (AUC). Logistic regression assumptions and model diagnostics were also assessed.

**Results:**

Radius, texture, smoothness, and concavity were significant predictors of malignancy. Five‐fold cross‐validation indicated stable model performance. On the test set, logistic regression achieved the highest accuracy (95.3%) and AUC (0.983), followed by SVM (94.1%, AUC = 0.980) and random forest (92.9%, AUC = 0.979). Additional performance metrics, including precision, F1‐score, and MCC, also demonstrated strong classification performance. Diagnostic analyses confirmed acceptable model fit and no major violations of logistic regression assumptions. No significant differences in AUC were observed among the models (DeLong test, *p* > 0.05).

**Conclusion:**

The study demonstrates that an integrated statistical and machine learning approach provides a robust and accurate method for breast cancer classification. Logistic regression showed slightly better performance, whereas machine learning models also achieved comparable results. This approach has strong potential to support early detection and clinical decision‐making.

## 1. Introduction

Breast cancer remains one of the most prevalent malignancies and a leading cause of cancer‐related mortality among women worldwide. According to the World Health Organization (WHO), approximately 2.3 million women were diagnosed with breast cancer and about 670,000 deaths occurred globally in 2022, highlighting its substantial public health burden [1]. Projections further indicate that the global incidence of breast cancer may exceed 3 million cases annually by 2040–2050, with a disproportionate increase expected in low‐ and middle‐income countries [2]. This rising burden underscores the urgent need for improved early detection, accurate diagnosis, and effective intervention strategies.

Breast cancer incidence has shown a consistent upward trend across many regions. Reports from the American Cancer Society indicate increasing rates, particularly for early‐stage and hormone receptor–positive cancers [3]. Additionally, global epidemiological studies reveal marked disparities in incidence and outcomes across populations, largely driven by differences in lifestyle factors, environmental exposures, and access to healthcare services [4]. These disparities highlight the importance of context‐specific research to better understand determinants of breast cancer and inform targeted prevention and control strategies.

Tumor morphology plays a critical role in distinguishing malignant from benign breast lesions. Advances in diagnostic techniques, particularly fine needle aspiration (FNA) combined with digital image analysis, have enabled the extraction of quantitative features such as radius, texture, perimeter, and concavity [5]. These features capture tumor heterogeneity and structural irregularities, which are strongly associated with malignancy and disease progression. The availability of high‐quality datasets derived from such techniques has facilitated the application of statistical and computational approaches for cancer classification.

In recent years, both statistical modeling and machine learning techniques have been increasingly applied to breast cancer diagnosis. Logistic regression remains a widely used and interpretable method for analyzing binary outcomes, offering clinically meaningful measures such as odds ratios (ORs) to quantify associations between predictors and outcomes [6]. However, machine learning algorithms, including random forest and support vector machine (SVM), provide complementary advantages by capturing complex and potentially nonlinear relationships among variables, thereby improving predictive performance [7, 8]. Several studies have demonstrated the effectiveness of these approaches in breast cancer classification tasks [9, 10].

Despite these advancements, many existing studies primarily focus on predictive accuracy while overlooking essential aspects of statistical modeling, such as multicollinearity assessment, goodness of fit evaluation, and residual diagnostics, which are critical for ensuring model validity and reliability [11]. Furthermore, limited studies provide a direct comparison between traditional statistical models and machine learning approaches within a unified analytical framework.

Therefore, this study is aimed at identifying the determinants of malignant breast tumors using a multivariable logistic regression model based on tumor morphological features derived from the Breast Cancer Wisconsin Diagnostic Dataset. In addition, machine learning models, including random forest and SVM, were implemented to compare predictive performance. The analysis incorporates comprehensive model diagnostics, including multicollinearity assessment, goodness of fit testing, and evaluation of discriminatory ability using the receiver operating characteristic (ROC) curve. This integrated approach enhances both the interpretability and predictive robustness of the findings, contributing to improved modeling practices in biomedical research.

## 2. Methods and Materials

### 2.1. Study Setting

This study utilized the publicly available Breast Cancer Wisconsin Diagnostic Dataset, hosted on the Kaggle platform. The dataset contains quantitative measurements of breast tumor cell nuclei obtained from FNA images. These data were originally collected in a clinical diagnostic setting to support accurate differentiation between benign and malignant tumors. The use of this dataset provides access to high‐quality, real‐world clinical measurements without requiring new patient recruitment.

### 2.2. Study Design

A retrospective secondary data analysis design was employed to examine the association between tumor morphological features and malignancy status. This approach enables robust statistical modeling using existing anonymized data and supports both explanatory and predictive analyses.

### 2.3. Study Population

The study population included all tumor samples contained in the dataset. Each observation represents a tumor with a confirmed diagnosis (malignant or benign) and corresponding morphological measurements. Both malignant and benign cases were included to ensure a comprehensive and unbiased analysis.

### 2.4. Sampling Technique and Sample Size

A census sampling approach was used, including all 569 observations available in the dataset. Records with missing or inconsistent values were excluded to maintain data quality. The use of the full dataset enhances statistical power and minimizes sampling bias.

### 2.5. Data Collection and Preprocessing

The original data were obtained through FNA procedures followed by digital image analysis to extract quantitative tumor features. For this study, the dataset was downloaded from Kaggle and preprocessed prior to analysis. Data cleaning procedures included checking for missing values, verifying variable consistency, and assessing variable distributions to identify potential outliers. Feature scaling and normalization were considered where appropriate to ensure compatibility with machine learning algorithms.

### 2.6. Study Variables

The outcome variable was tumor diagnosis, coded as a binary variable (1 = malignant and 0 = benign). The explanatory variables consisted of eight continuous tumor morphological features: *radius_mean, texture_mean, perimeter_mean, area_mean, smoothness_mean, compactness_mean, concavity_mean,* and *concave_points_mean*. These predictors were selected based on their established diagnostic relevance in breast cancer research and their ability to represent key tumor characteristics, including size, texture, shape, and boundary irregularity. Previous studies have identified these morphological features as important indicators of malignancy and useful predictors for breast cancer classification. Restricting the analysis to these biologically meaningful variables enhanced model interpretability while reducing redundancy among highly correlated features within the original dataset.

### 2.7. Statistical Analysis

#### 2.7.1. Logistic Regression Model

The primary analytical approach was multivariable logistic regression, which is appropriate for modeling binary outcome variables. The model estimates the log‐odds of malignancy as a linear function of the explanatory variables:

logp1−p=β0+β1X1+β2X2+⋯+βkXk,

where *p* is the probability that a tumor is malignant, *X*_1_, *X*_2_, ⋯*X*_*k*_ are the predictor variables, *β*_0_ is the intercept, and *β*_1_, *β*_2_, ⋯*β*_*k*_ are the regression coefficients representing the change in the log‐odds of malignancy per unit change in the predictor. The estimated coefficients were exponentiated to obtain ORs with 95% confidence intervals, providing interpretable measures of association [12, 13]. Logistic regression remains a fundamental method in medical research due to its interpretability and solid theoretical foundation [14].

#### 2.7.2. Machine Learning Models

To complement the logistic regression analysis, two machine learning models were implemented. Random forest, an ensemble‐based method, constructs multiple decision trees and aggregates their predictions to improve classification accuracy and robustness [15]. SVM identifies an optimal decision boundary in high‐dimensional space and is particularly effective for classification problems involving complex data structures [16]. These models have demonstrated strong performance in biomedical classification tasks, particularly when nonlinear relationships exist among predictors [17].

#### 2.7.3. Model Performance Evaluation

The dataset was partitioned into training (70%) and testing (30%) subsets using stratified random sampling to preserve the distribution of benign and malignant cases. The same training and testing sets were used for logistic regression, random forest, and SVM models to ensure a fair comparative evaluation. Furthermore, five‐fold cross‐validation was performed on the training dataset to assess model robustness and generalizability. Hyperparameter tuning was conducted within the cross‐validation framework, with the optimal parameters subsequently applied to the final random forest and SVM models.

Model performance was assessed using accuracy, sensitivity, specificity, and the area under the curve (AUC). The ROC curve is a widely used tool for evaluating classification models and provides a threshold‐independent measure of discrimination [18]. Higher AUC values indicate better model performance in distinguishing between malignant and benign tumors. The DeLong test was applied to statistically compare AUC values between models [19].

#### 2.7.4. Model Diagnostics

Diagnostic procedures were conducted to assess the validity of the logistic regression model. Multicollinearity was evaluated using the variance inflation factor (VIF), with higher values indicating potential collinearity issues [20]. Model calibration and goodness of fit were assessed using the Hosmer–Lemeshow test [6]. Residual diagnostics, including standardized residuals, deviance residuals, and Cook′s distance, were examined to identify influential observations and potential model violations [21].

All analyses were conducted using R statistical software [22], a widely used environment for statistical computing and machine learning. Relevant packages were utilized for regression modeling, machine learning algorithms, and performance evaluation [23]. A significance level of *α* = 0.05 was used for all statistical tests.

## 3. Results

### 3.1. Descriptive Results

Table 1 summarizes the descriptive statistics of tumor morphological features for the overall sample and by diagnosis. A total of 569 tumor samples were analyzed, including 357 benign and 212 malignant cases. Overall, malignant tumors exhibited consistently higher mean values across all features compared with benign tumors, indicating more aggressive characteristics. In particular, malignant tumors showed substantially larger size‐related measures, including radius (17.5 ± 3.2 vs. 12.1 ± 1.8), perimeter (115 ± 22 vs. 78 ± 12), and area (978 ± 368 vs. 463 ± 134). Texture was also higher among malignant tumors (21.6 ± 3.8) compared with benign tumors (17.9 ± 4.0), reflecting greater heterogeneity. Furthermore, measures of tumor shape irregularity, including compactness (0.15 ± 0.05 vs. 0.08 ± 0.03), concavity (0.16 ± 0.08 vs. 0.05 ± 0.04), and concave points (0.09 ± 0.03 vs. 0.03 ± 0.02), were markedly elevated in malignant cases, indicating more irregular and invasive tumor boundaries. Smoothness showed a smaller difference between groups (0.103 ± 0.013 vs. 0.092 ± 0.013). Overall, these findings demonstrate that malignant tumors are characterized by larger size, increased heterogeneity, and greater structural irregularity compared with benign tumors, supporting the relevance of these features for breast cancer classification.

**Table 1:** Descriptive statistics of tumor morphological features stratified by breast cancer diagnosis.

Characteristic	Overall (*N* = 569)^a^	B (*N* = 357)^a^	M (*N* = 212)^a^
radius_mean	14.1 ± 3.5	12.1 ± 1.8	17.5 ± 3.2
texture_mean	19.3 ± 4.3	17.9 ± 4.0	21.6 ± 3.8
perimeter_mean	92 ± 24	78 ± 12	115 ± 22
area_mean	655 ± 352	463 ± 134	978 ± 368
smoothness_mean	0.096 ± 0.014	0.092 ± 0.013	0.103 ± 0.013
compactness_mean	0.10 ± 0.05	0.08 ± 0.03	0.15 ± 0.05
concavity_mean	0.09 ± 0.08	0.05 ± 0.04	0.16 ± 0.08
concave_points_mean	0.05 ± 0.04	0.03 ± 0.02	0.09 ± 0.03

^a^Mean ± SD.

### 3.2. Multivariable Logistic Regression Analysis

In Table 2, the multivariable logistic regression analysis identified several tumor morphological features significantly associated with breast cancer malignancy. Specifically, radius_mean (*β* = 1.01, *p* < 0.001) and texture_mean (*β* = 0.34, *p* < 0.001) were strong positive predictors, indicating that tumors with larger size and greater texture variation have higher odds of being malignant. Similarly, smoothness_mean (*β* = 80.63, *p* = 0.043) and concavity_mean (*β* = 26.86, *p* = 0.040) were also statistically significant, suggesting that irregular tumor surfaces and greater concave structures are associated with malignancy. In contrast, compactness_mean (*p* = 0.322) and concave_points_mean (*p* = 0.457) were not statistically significant, indicating limited independent contribution after adjusting for other variables. Overall, the results demonstrate that tumor size, texture, and shape irregularity are key determinants of breast cancer malignancy, although some predictors exhibit large coefficient estimates, which may reflect scaling issues or residual variability in the data.

**Table 2:** Multivariable logistic regression (final model).

Variable	*β*(estimate)	SE	*p* value
Intercept	−31.27	6.69	< 0.001 ^∗^
radius_mean	1.01	0.24	< 0.001 ^∗^
texture_mean	0.34	0.07	< 0.001 ^∗^
smoothness_mean	80.63	39.88	0.043 ^∗^
compactness_mean	−12.84	12.97	0.322
concavity_mean	26.86	13.05	0.040 ^∗^
concave_points_mean	24.58	33.07	0.457

∗Statistical significance at *p* < 0.05.

Multicollinearity among the explanatory variables in the final multivariable logistic regression model was assessed using the VIF. As shown in Table 3, all VIF values were below the threshold of 10, indicating no evidence of severe multicollinearity. The VIF values ranged from 5.16 to 6.10, with moderate levels observed for smoothness_mean, concavity_mean, and concave_points_mean. These values are within acceptable limits and do not adversely affect the stability or interpretability of the model. Therefore, all variables were retained in the final analysis.

**Table 3:** Variance inflation factors (VIFs) for predictors in the final model.

Variable	VIF
radius_mean	2.66
texture_mean	1.52
smoothness_mean	5.16
compactness_mean	4.23
concavity_mean	5.43
concave.points_mean	6.10

As shown in Table 4, logistic regression diagnostics indicated satisfactory model adequacy. VIF values ranged from 1.52 to 6.10, suggesting acceptable levels of multicollinearity among predictors. The Hosmer–Lemeshow goodness of fit test demonstrated excellent calibration of the model (*p* = 0.9974). The maximum Cook′s distance was 0.4285, indicating the absence of highly influential observations. Standardized residuals ranged from −2.65 to 2.76, with no observations exceeding the conventional threshold of ± 3. The Box–Tidwell test was not statistically significant (*p* = 0.56), indicating that the assumption of linearity between continuous predictors and the logit of the outcome was satisfied. Overall, these findings support the adequacy and stability of the fitted logistic regression model.

**Table 4:** Logistic regression diagnostic assessment.

Diagnostic	Result
VIF range	1.52–6.10
Hosmer–Lemeshow *p* value	0.9974
Maximum Cook′s distance	0.4285
Maximum standardized residual	2.76
Box–Tidwell *p* value	0.56

In Table 5, the logistic regression model demonstrated excellent classification performance, with an accuracy of 95.3%, sensitivity of 92.1%, and specificity of 97.2%. Furthermore, the model exhibited outstanding discriminatory ability, as indicated by an AUC of 0.983.

**Table 5:** Performance of the logistic regression model in breast cancer classification.

Model	Accuracy	Sensitivity	Specificity	AUC
Logistic regression	0.953	0.921	0.972	0.983

**Figure 1:**
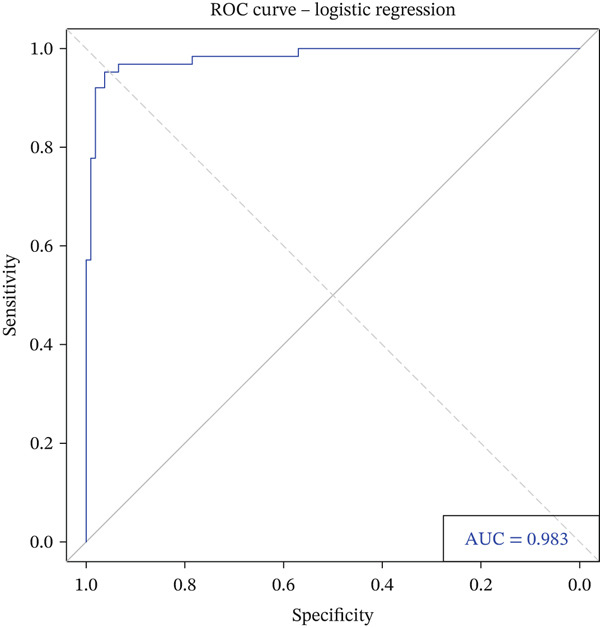
ROC curve demonstrating the diagnostic performance of the logistic regression model.

Figure 1 shows, the ROC curve demonstrates that the logistic regression model performs very well in distinguishing between outcome categories, as evidenced by the curve′s proximity to the upper left corner. This indicates high sensitivity across a range of specificity values, reflecting excellent overall discriminative ability and performance well above chance.

### 3.3. Random Forest Model Results

To assess the robustness and generalizability of the developed classification models, five‐fold cross‐validation was performed using the training dataset. As shown in Table 6, the SVM achieved the highest mean cross‐validation accuracy (0.9449 ± 0.0287), followed by the random forest model (0.9398 ± 0.0165) and logistic regression (0.9273 ± 0.0242). The relatively small standard deviations across folds indicate consistent predictive performance and suggest that the models were not substantially influenced by sampling variability. These findings demonstrate the stability of the developed models and support the reliability of the subsequent test‐set performance evaluation.

**Table 6:** Five‐fold cross‐validation performance of the evaluated models.

Model	Mean CV accuracy	SD
Logistic regression	0.9273	0.0242
Random forest	0.9398	0.0165
Support vector machine	0.9449	0.0287

Comparison of cross‐validation and independent test‐set performance revealed only minor differences in classification accuracy across all models, suggesting limited evidence of overfitting.

Hyperparameter tuning was conducted within the five‐fold cross‐validation framework to optimize model performance. As shown in Table 7, the random forest model achieved optimal performance with mtry = 4, indicating that four predictor variables were randomly considered at each split. For the SVM with a radial basis function (RBF) kernel, the optimal parameters were sigma = 0.2419 and *C* = 2. These tuned values were subsequently used to develop the final models evaluated on the independent test dataset.

**Table 7:** Optimal hyperparameters obtained from five‐fold cross‐validation.

Model	Hyperparameter	Optimal value
Random forest	mtry	4
Support vector machine	Sigma	0.2419
Support vector machine	Cost (*C*)	2

The performance of the random forest classifier is presented in Table 8, with the corresponding confusion matrix shown in Table 9. Out of 170 test samples, 159 were correctly classified, yielding an overall accuracy of 93.5%. The model correctly identified 101 benign and 58 malignant cases, whereas 11 misclassifications were observed, consisting of 6 false positives and 5 false negatives. In addition, the model achieved a sensitivity of 92.06%, indicating its ability to correctly identify malignant cases, and a specificity of 94.39%, reflecting its performance in correctly classifying benign cases. Overall, the random forest model demonstrates strong classification performance with high accuracy and a balanced ability to distinguish between benign and malignant cases.

**Table 8:** Performance of random forest model for breast cancer classification.

Metric/outcome	Value
Total test samples	170
Correctly classified (benign)	101
Correctly classified (malignant)	58
False positive (benign → malignant)	6
False negative (malignant → benign)	5
Total correct predictions	159
Accuracy	93.5%
Sensitivity (malignant detection)	92.06%
Specificity (benign detection)	94.39%

**Table 9:** Confusion matrix of random forest model.

Predicted/actual	Benign (B)	Malignant (M)
Benign (B)	101	5
Malignant (M)	6	58

**Figure 2:**
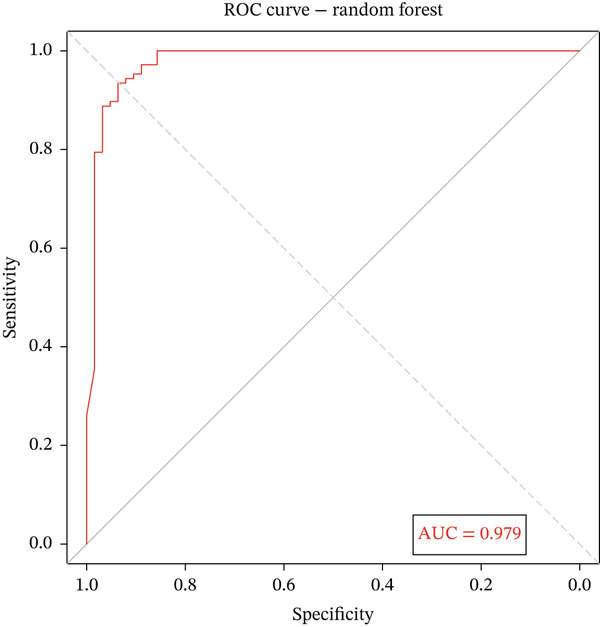
Receiver operating characteristic (ROC) curve for the random forest model in breast cancer classification.

Figure 2 shows that the ROC curve for the random forest model shows strong classification performance, as the curve is positioned close to the upper left corner of the plot. This indicates that the model achieves high sensitivity while maintaining high specificity, reflecting an effective balance between correctly identifying positive cases and minimizing false positives. The curve lies well above the diagonal reference line, confirming that the model performs substantially better than random classification. Overall, the shape of the curve suggests excellent discriminative ability.

**Figure 3:**
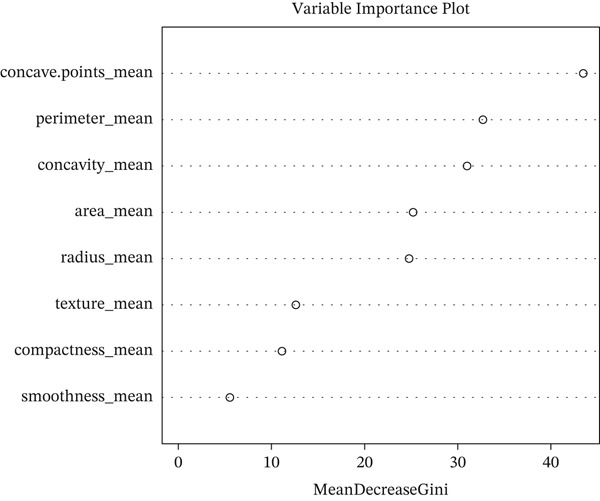
Random forest variable importance plot.

Variable importance analysis revealed that concave_points_mean was the most influential predictor in the random forest model, followed by perimeter_mean and concavity_mean (Figure 3). Variables associated with tumor size and shape irregularity, including area_mean and radius_mean, also demonstrated substantial predictive importance. Conversely, smoothness_mean, compactness_mean, and texture_mean contributed less to the classification process. These findings suggest that morphological features reflecting tumor boundary irregularity and size play a critical role in distinguishing malignant from benign breast tumors. This analysis enhances the interpretability of the machine learning model by identifying the predictors that contributed most strongly to classification performance.

### 3.4. SVM Results

In Table 10, the SVM model was trained using a RBF kernel with a cost parameter of 1, and it identified a total of 90 support vectors, comprising 49 from the benign class and 41 from the malignant class. Support vectors represent the most influential observations that define the optimal decision boundary between classes; therefore, the moderate number observed suggests that the model effectively captured the underlying structure of the data without depending on the entire dataset, thereby enhancing its generalization ability. The use of the RBF kernel enabled the model to accommodate potential nonlinear relationships between predictor variables and the diagnosis outcome, making it well‐suited for complex biomedical datasets such as breast cancer classification. Furthermore, the presence of two distinct classes (benign and malignant) confirms that the model was appropriately formulated as a binary classification framework, consistent with diagnostic prediction objectives.

**Table 10:** Support vector machine (SVM) model summary.

Item	Result
Model type	C‐classification
Kernel used	Radial basis function (RBF)
Cost parameter (*C*)	1
Number of support vectors	90
Support vectors (Class B)	49
Support vectors (Class M)	41
Number of classes	2
Class labels	Benign (B), malignant (M)

The SVM model demonstrated strong classification performance in distinguishing between benign and malignant cases of breast cancer. As presented in Tables 11 and 12, out of 170 test observations, the model correctly classified 160 cases, yielding an overall accuracy of 94.12%. Table 11 shows that 103 benign and 57 malignant cases were correctly identified, whereas 10 cases were misclassified, including 6 malignant cases incorrectly predicted as benign (false negatives) and 4 benign cases incorrectly predicted as malignant (false positives). The summary in Table 12 further confirms the model′s high performance, reporting a low misclassification rate consistent with the confusion matrix results. From a clinical perspective, the presence of false negatives is particularly critical, as these represent missed cancer cases that may delay diagnosis and treatment. Nevertheless, the overall low error rate across Tables 11 and 12 indicates that the SVM model provides reliable and robust predictive performance for breast cancer classification and is suitable for supporting diagnostic decision‐making.

**Table 11:** Confusion matrix of SVM model for breast cancer classification.

Predicted/actual	Benign (B)	Malignant (M)
Benign (B)	103	6
Malignant (M)	4	57

**Table 12:** Performance summary of SVM model for breast cancer classification.

Metric	Value
Total test samples	170
Correctly classified	160
Misclassified cases	10
Accuracy	94.12%

**Figure 4:**
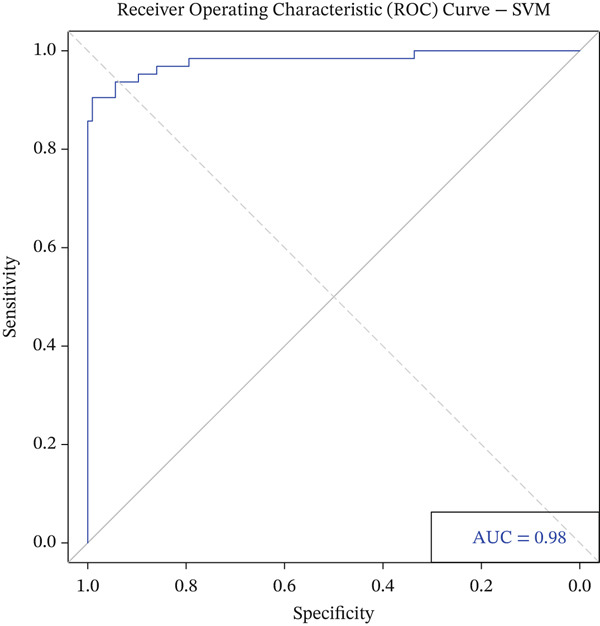
Receiver operating characteristic (ROC) curve for SVM model performance.

In Figure 4, the ROC curve illustrates the diagnostic performance of the SVM model in distinguishing between benign and malignant cases. The model achieved an AUC of 0.98, indicating excellent discriminative ability. The curve lies close to the top‐left corner, demonstrating high sensitivity and specificity across different classification thresholds.

### 3.5. Model Comparison

As shown in Table 13, all three models demonstrated excellent classification performance, with AUC values exceeding 0.97. Logistic regression achieved the highest overall performance, with an accuracy of 0.953, specificity of 0.972, precision of 0.951, F1‐score of 0.935, Matthews correlation coefficient (MCC) of 0.899, and an AUC of 0.983 (95% CI: 0.966–1.000). Random forest achieved an accuracy of 0.929 and an AUC of 0.979 (95% CI: 0.955–1.000), whereas SVM attained an accuracy of 0.941 and an AUC of 0.980 (95% CI: 0.958–1.000). Although all models showed high sensitivity (0.905–0.921) and specificity (0.935–0.972), logistic regression consistently outperformed the machine learning models across most evaluation metrics. These findings indicate that logistic regression provided the most accurate and balanced classification of benign and malignant breast tumors in the present study.

**Table 13:** Comparative performance of logistic regression, random forest, and support vector machine models.

Model	Accuracy	Sensitivity	Specificity	Precision	F1‐score	MCC	AUC (95% CI)
Logistic regression	0.953	0.921	0.972	0.951	0.935	0.899	0.983 (0.966–1.000)
Random forest	0.929	0.921	0.935	0.892	0.906	0.850	0.979 (0.955–1.000)
Support vector machine	0.941	0.905	0.963	0.934	0.919	0.873	0.980 (0.958–1.000)

In Table 14, the DeLong test revealed no statistically significant differences in the AUCs among the three models. Although logistic regression showed a slightly higher AUC, all pairwise comparisons indicated *p* values greater than 0.05, suggesting comparable discriminatory performance between logistic regression, random forest, and SVM.

**Table 14:** Pairwise comparison of AUCs using DeLong′s test.

Model comparison	AUC (Model 1)	AUC (Model 2)	*Z*‐value	*p* value
Logistic regression versus RF	0.983	0.978	0.767	0.443
Logistic regression versus SVM	0.983	0.980	0.490	0.624
Random forest versus SVM	0.978	0.980	−0.642	0.521

**Figure 5:**
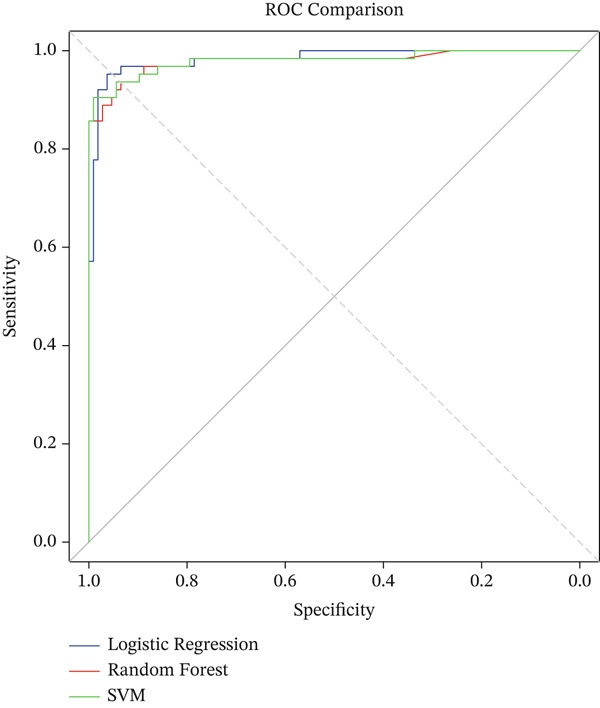
ROC curve performance comparison of logistic regression, random forest, and SVM for breast cancer classification.

In Figure 5, the ROC curve analysis showed that all three models (logistic regression, random forest, and SVM) achieved high discriminatory power, with curves approaching the upper left corner of the plot. SVM and logistic regression exhibited slightly superior performance compared with random forest, although differences were minimal, indicating robust predictive capability across all models.

## 4. Discussion

This study developed an integrated statistical and machine learning framework for breast cancer classification using tumor morphological features, demonstrating high predictive performance and strong discriminative capability between benign and malignant tumors. These findings are consistent with a growing body of recent evidence indicating that machine learning and deep learning models can achieve highly accurate and robust performance in breast cancer diagnosis [24, 25]. In particular, recent studies emphasize that integrating feature selection and advanced learning algorithms significantly enhances classification accuracy and model stability [26].

The strong predictive performance observed in this study can largely be attributed to the discriminative power of tumor morphological features. Features such as radius, texture, perimeter, and concavity capture critical structural characteristics of tumors and have been consistently identified as key predictors of malignancy. These findings align with recent literature demonstrating that morphological and radiomic features provide essential information for distinguishing between benign and malignant lesions [27, 28]. Biologically, malignant tumors exhibit irregular shapes, heterogeneous cellular architecture, and invasive growth patterns, which are effectively captured through these features and contribute to improved classification performance.

Some predictors, particularly smoothness_mean and concavity_mean, exhibited relatively large coefficient estimates in the logistic regression model. This finding may be attributable to differences in measurement scales and the strong discriminatory power of these variables [20]. Although moderate correlations existed among some morphological features, diagnostic evaluation indicated no evidence of severe multicollinearity or coefficient instability, with VIF values remaining within acceptable limits. Furthermore, model diagnostic assessments, including the Hosmer–Lemeshow goodness of fit test, residual analysis, and influence diagnostics, demonstrated excellent calibration and stable predictive performance. These findings suggest that the observed coefficient estimates reflect meaningful associations with malignancy rather than numerical artifacts arising from model misspecification.

A key contribution of this study is the integration of statistical modeling with machine learning techniques, which provides both predictive accuracy and interpretability. Although machine learning models are often criticized for their “black‐box” nature, the inclusion of statistical inference enables the identification of significant predictors and improves transparency. This approach is consistent with recent advancements in explainable artificial intelligence, which emphasize the importance of interpretability for clinical adoption [29, 30]. Moreover, emerging research highlights that combining statistical and machine learning frameworks can enhance both model performance and clinical trust [31].

From a methodological perspective, the comparative performance of machine learning algorithms underscores the importance of model selection in biomedical applications. Recent studies confirm that ensemble and hybrid approaches outperform individual classifiers by effectively capturing nonlinear relationships and reducing overfitting [32, 33]. Additionally, modern frameworks incorporating deep learning and hybrid architectures have demonstrated superior performance in breast cancer classification tasks [25]. These findings support the robustness of the integrated modeling approach used in this study. The consistency between cross‐validation and independent test‐set performance further suggests that the developed models generalized well and exhibited limited evidence of overfitting. To mitigate multicollinearity, highly correlated variables were evaluated using VIFs. Variables exhibiting excessive collinearity were excluded during model development, resulting in acceptable VIF values ranging from 1.52 to 6.10 in the final logistic regression model. These findings indicate that multicollinearity was adequately controlled and that the estimated regression coefficients are likely to be stable and interpretable.

The high discriminative ability of the model, as reflected by evaluation metrics such as accuracy and AUC, further supports its potential clinical utility. The high classification performance observed across all models suggests potential utility as decision‐support tools for assisting clinicians in the early identification of malignant breast lesions. Such models may complement existing diagnostic workflows, improve diagnostic consistency, and contribute to more timely clinical decision‐making. AUC remains a widely accepted measure of diagnostic performance, capturing the balance between sensitivity and specificity. Recent studies also emphasize the importance of complementary evaluation metrics, such as the MCC, particularly in handling class imbalance [34]. The strong performance observed in this study is consistent with recent meta‐analyses reporting high diagnostic accuracy of machine learning models across various imaging and clinical datasets [28, 35].

Recent deep learning and hybrid frameworks, including enhanced exception networks, vision transformers, EfficientNet‐based architectures, and stacking ensemble models, have demonstrated excellent performance in cancer diagnosis and medical image classification [36–39]. However, these approaches often require larger datasets, substantial computational resources, and reduced interpretability. In contrast, the present study achieved excellent discrimination (AUC > 0.97) using interpretable logistic regression, random forest, and SVM models based on a limited set of tumor morphological features, highlighting their potential utility in resource‐constrained clinical settings.

Despite these promising findings, several limitations should be acknowledged. First, the reliance on a single publicly available dataset may limit the generalizability of the findings to other populations and clinical settings. Variations in patient characteristics, imaging procedures, and data quality may influence model performance when applied externally. Therefore, external validation using independent datasets and real‐world clinical populations is required before routine clinical implementation of the proposed models. Although variable selection techniques such as LASSO and stepwise regression were not applied, predictor selection was guided by biological relevance, prior evidence, and multicollinearity assessment. Future studies may investigate automated feature selection approaches to further improve model parsimony and predictive efficiency. In addition, precision–recall curve analysis was not performed and may provide complementary insights into classification performance, particularly in settings with class imbalance. Second, although morphological features provide valuable diagnostic information, they do not fully capture the molecular and genetic heterogeneity of breast cancer. Future research should investigate the integration of morphological, clinical, genomic, and imaging data to further enhance diagnostic accuracy and clinical applicability. Recent studies suggest that multimodal approaches can substantially improve predictive performance and support more personalized clinical decision‐making [40, 41].

Furthermore, translating machine learning models into real‐world clinical practice requires careful validation and consideration of implementation challenges. These include issues related to data privacy, model interpretability, and integration into healthcare systems. Emerging approaches such as federated learning offer promising solutions by enabling collaborative model development while preserving data privacy [42]. Addressing these challenges is essential for ensuring the safe and effective deployment of machine learning models in clinical settings.

## 5. Conclusion and Recommendation

### 5.1. Conclusion

This study demonstrated that an integrated statistical and machine learning approach can effectively classify breast tumors using a limited set of morphological features. Using a stratified 70:30 train–test split, five‐fold cross‐validation, and hyperparameter optimization, logistic regression, random forest, and SVM models all achieved excellent predictive performance. Logistic regression showed the highest overall performance, with an accuracy of 95.3% and an AUC of 0.983, whereas random forest and SVM also demonstrated strong discrimination, with AUC values exceeding 0.97. Cross‐validation results further indicated stable model performance and limited evidence of overfitting.

The selected morphological features were highly informative for distinguishing malignant from benign tumors, and logistic regression identified several significant predictors of malignancy. Model diagnostic assessments confirmed acceptable multicollinearity levels, good calibration, and stable coefficient estimates, supporting the validity of the statistical model. Overall, the findings suggest that combining interpretable statistical modeling with machine learning techniques provides a robust framework for breast cancer classification and may serve as a useful decision‐support tool for early diagnosis. Future studies should validate these findings using independent clinical datasets and explore the integration of clinical, genomic, and imaging information to further enhance predictive performance and clinical applicability.

### 5.2. Recommendations

Based on the findings of this study, future research should validate the developed models using independent datasets and real‐world clinical populations to assess their generalizability and applicability across different healthcare settings. The integration of additional data sources, including clinical, genomic, and imaging information, may further improve diagnostic accuracy and clinical relevance.

Future studies may also explore automated feature selection techniques, such as LASSO and stepwise regression, to enhance model parsimony and identify the most informative predictors. In addition, advanced machine learning and deep learning approaches, including hybrid and explainable artificial intelligence frameworks, should be investigated and compared with conventional models to further improve predictive performance and interpretability.

From a clinical perspective, the high classification performance observed across all models suggests their potential utility as decision‐support tools for assisting clinicians in the early detection of breast cancer. However, comprehensive external validation and prospective evaluation are required before routine clinical implementation.

## Author Contributions

Awoke Fetahi Woudneh conceived and designed the study, performed the data analysis, interpreted the results, and drafted the manuscript. Nigatu Tiruneh Shiferaw contributed to data analysis, interpretation of results, and critically reviewed the manuscript for important intellectual content. Yenesew Fentahun Gebrie participated in study design, data interpretation, and manuscript revision. Misganaw Mekonnen Nigussie contributed to data analysis, interpretation, and manuscript editing. Kebadu Tadesse Cherie contributed to data interpretation and manuscript revision. Fetene Getnet Gebeyehu contributed to data interpretation and manuscript editing.

## Funding

No funding was received for this manuscript.

## Disclosure

All authors read and approved the final manuscript. The manuscript has not been published elsewhere and is not under consideration for publication in any other journal. The authors agree to submit this manuscript to this journal for publication as original research.

## Ethics Statement

This study used a publicly available secondary dataset (Breast Cancer Wisconsin Diagnostic Dataset) obtained from Kaggle. The dataset contains anonymized records and does not include identifiable personal information. Therefore, ethical approval and informed consent were not required.

## Consent

This study used a publicly available, de‐identified dataset. Therefore, informed consent and consent to publish were not applicable.

## Conflicts of Interest

The authors declare no conflicts of interest.

## Data Availability

The dataset used in this study is publicly available from Kaggle: https://www.kaggle.com/datasets/erdemtaha/cancer-data.
